# Bienvenida a Archivos Peruanos de Cardiología y Cirugía Cardiovascular

**DOI:** 10.47487/apcyccv.v1i1.3

**Published:** 2020-03-30

**Authors:** Manuel Alberto Chacón Diaz

**Affiliations:** Editor General, Archivos Peruanos de Cardiología y Cirugía Cardiovascular Archivos Peruanos de Cardiología y Cirugía Cardiovascular

El conocimiento científico, en particular en el área de la salud cardiovascular, está constantemente en evolución a través de la generación permanente de artículos científicos a nivel mundial. En nuestro país, la historia médica nos muestra lo valiosa que puede ser la investigación científica para dar respuesta a los problemas de salud de la población, así Daniel Alcides Carrión, Hermilio Valdizán, Alberto Hurtado, Carlos Monge y otros prestigiosos investigadores médicos han llevado a la medicina peruana a tener reconocimiento a nivel regional y mundial.

En el ámbito de la cardiología nacional, muchas veces, los intentos de investigación se han visto relegados por el desconocimiento de metodologías para la investigación, falta de grupos de investigación dedicados a tiempo completo para este fin, así como la falta de un medio de difusión local (físico o virtual), bien estructurado, que cumpla con las expectativas de los usuarios y las recomendaciones de los editores de revistas a nivel mundial.

Es así que **Archivos Peruanos de Cardiología y Cirugía Cardiovascular** nace con la finalidad de ser ese medio de difusión científica nacional de calidad, en el cual se vea reflejada las experiencias en el campo cardiovascular de los profesionales de salud en el Perú (médicos, enfermeras, tecnólogos, químico-farmacéuticos, etc), cubra la necesidad de información adecuada sobre las características de prevención, presentación y tratamiento de las patologías cardiovasculares que aquejan a nuestra sociedad y logre la indexación en las más prestigiosas bases de datos bibliográficos (Scielo, Pubmed, Scopus). Para este fin, contamos con destacados profesionales con experiencia en investigación de diversos establecimientos de salud a nivel nacional, quienes conforman el consejo editorial de la revista y de profesionales de altísimo reconocimiento a nivel internacional quienes son parte del consejo consultivo. No podríamos lograr estas metas sin el soporte del Instituto Nacional Cardiovascular INCOR de Essalud como entidad editora.

Cada número contará con artículos de revisión, artículos originales, reporte de casos, imágenes en patología cardiovascular y cartas al editor, con lo que esperamos mejorar la calidad del acceso a la información nacional e internacional de nuestros lectores. El proceso editorial de estos artículos cumple los requerimientos mínimos para hacer de la revista una publicación científica de calidad a través del sistema Open Journal System (OJS) y con evaluación por pares de gran experiencia y reconocimiento nacional e internacional.

El consejo editorial agradece a todos los profesionales de salud que han contribuido con sus artículos para la realización de este primer número y en especial al Profesor Thomas Lüscher por su invalorable aporte al realizar el primer editorial de la revista.

Sin más preámbulo y a nombre del consejo editorial, les doy la más calurosa bienvenida a esta, SU REVISTA.

